# Identifying the Driving Factors of Black Bloom in Lake Bay through Bayesian LASSO

**DOI:** 10.3390/ijerph16142492

**Published:** 2019-07-12

**Authors:** Liang Wang, Yulin Wang, Haomiao Cheng, Jilin Cheng

**Affiliations:** 1 School of Hydraulic Energy and Power Engineering, Yangzhou University, 196, Huayang Xi Road, Yangzhou 225127, China; 2 School of Environmental Science and Engineering, Yangzhou University, 196, Huayang Xi Road, Yangzhou 225127, China

**Keywords:** black bloom, Fe(II), S(−II), SSC, aquatic factors, Bayesian LASSO method, uncertainty analysis

## Abstract

Black blooms are a serious and complex problem for lake bays, with far-reaching implications for water quality and drinking safety. While Fe(II) and S(−II) have been reported as the most important triggers of this phenomenon, little effort has been devoted in investigating the relationships between Fe(II) and S(−II) and the host of potentially important aquatic factors. However, a model involving many putative predictors and their interactions will be oversaturated and ill-defined, making ordinary least squares (OLS) estimation unfeasible. In such a case, sparsity assumption is typically required to exclude the redundant predictors from the model, either through variable selection or regularization. In this study, Bayesian least absolute shrinkage and selection operator (LASSO) regression was employed to identify the major influence variables from 11 aquatic factors for Fe(II), S(−II), and suspended sediment concentration (SSC) in the Chaohu Lake (Eastern of China) bay during black bloom maintenance. Both the main effects and the interactions between these factors were studied. The method successfully screened the most important variables from many items. The determination coefficients (*R*^2^) and adjusted determination coefficients (Adjust *R*^2^) showed that all regression equations for Fe(II), S(-II), and SSC were in good agreement with the situation observed in the Chaohu Lake. The outcome of correlation and LASSO regression indicated that total phosphorus (TP) was the single most important factor for Fe(II), S(-II), and SSC in black bloom with explanation ratios (ERs) of 76.1%, 37.0%, and 12.9%, respectively. The regression results showed that the interaction items previously deemed negligible have significant effects on Fe(II), S(−II), and SSC. For the Fe(II) equation, total nitrogen (TN) × dissolved oxygen (DO) and chlorophyll a (CHLA) × oxidation reduction potential (ORP), which contributed 10.6% and 13.3% ERs, respectively, were important interaction variables. TP emerged in each key interaction item of the regression equation for S(−II). Water depth (DEP) × Fe(II) (30.7% ER) was not only the main interaction item, but DEP (5.6% ER) was also an important single factor for the SSC regression equation. It also indicated that the sediment in shallow bay is an important source for SSC in water. The uncertainty of these relationships was also estimated by the posterior distribution and coefficient of variation (CV) of these items. Overall, our results suggest that TP concentration is the most important driver of black blooms in a lake bay, whereas the other factors, such as DO, DEP, and CHLA act in concert with other aquatic factors. There results provide a basis for the further control and management policy development of black blooms.

## 1. Introduction

Black blooms in lakes, generally occurring in bay or windward shore areas, are a sudden water blackening and stinking phenomenon. This extreme pollution phenomenon has frequently occurred since the early 1970s, leading to serious water quality deterioration [1]. While black blooms used to occur in lakes of developed countries, such as a strip-mine lake in the United States [2], the Lower Mystic Lake in the United States [3], and the Garda Lake in Italy [4], they have become a critical and recurring issue for developing countries such as China and other countries undergoing a rapid modernization period [5,6,7]. The frequent occurrence of black blooms in lakes seriously threatens the safe supply of drinking water for urban residents and restricts the development of regional economies. Therefore, effectively reducing the occurrence of black blooms in lakes is a water environmental problem that needs to be solved. Reducing and preventing the black blooms in lakes has attracted the attention of scholars and management departments at all levels.

Field and laboratory investigations have shown that lake black blooms are caused by an imbalance in biogeochemical processes [8]. The processes involved in lake black blooms can be summarized in three steps. First, the algae grow, accumulate, and remain in a shallow lake bay for a long period under weak wind and water dynamics conditions [9,10,11]. The death and rot of the gathered algae under high atmospheric temperature lead to a sharp decrease in the water dissolved oxygen (DO) and the water and sediment becoming anaerobic in the lake bay [9,12]. Second, under these conditions, the system moves from anoxic to euxinic. High valence iron and sulfur reduce to soluble ferrous, soluble inorganic sulfides, and volatile organic sulfide compounds in an anaerobic aquatic environment [13]. The algae decomposition supplies rich organic matter for this process [14]. Third, the wind waves bring anaerobic reaction products to the water surface and black blooms are observed [15]. The organic sulfide compounds, mainly dimethyl sulfides (DMSs) and H_2_S are the key odorous compounds in black bloom [16,17,18]. The soluble ferrous and soluble inorganic sulfides form FeS granules and cause the water to become seriously turbid. FeS granules are black and cloud the lake water so much that the water looks black [19]. This is the reason that this phenomenon is called “black bloom”.

Many investigations on the relationship between the total phosphorus (TP), total nitrogen (TN), DO, water color, and chlorophyll a (CHLA) in black bloom lakes have been reported [20,21,22] as applications of multivariate regression. Although soluble ferrous and soluble inorganic sulfides have been shown to be the limiting factors causing black bloom in lakes [23,24,25], the quantitative relationship between aquatic environmental factors has not received enough attention, except for DO and the water velocity of S(−II) and Fe(II) [26,27]. Fe(II) (S(−II)) is not only present as soluble ferrous (inorganic sulfides) but also as soluble ferrous (sulfides) plus ferrous (S(−II)) in inorganic compounds (such as FeS ), as the measurements are recorded under acidic conditions. The uncertainty in these relationships, which would be a key tool for governing and managing black bloom water, has not been examined. In many developing countries, Fe(II) and S(−II) are not routinely monitored pollutants, so the data are scarce. Black blooms in lakes occur suddenly and irregularly and are of short duration. This increases the difficulty of obtaining data about black bloom water. The Bayesian method, which is well-suited to deal with uncertainty in model parameters, is hard to use for screening important factors for Fe(II) and S(−II), especially with sparse data [28]. These difficulties impeded the detailed quantitative investigation of relationships among Fe(II), S(−II), and several aquatic factors and their interactions.

In this study, data on several environmental factors, including Fe(II) and S(−II) recorded from black bloom waters in Chaohu Lake (Eastern of China) are analyzed. Bayesian LASSO (least absolute shrinkage and selection operator) [29,30] was used to screen potential predictors of Fe(II), S(−II), and the suspended sediment concentration(SSC), considering both main and interaction effects of predictors. While interaction effects have seldom been analyzed before, their identification may provide valuable clues for informed decision-making with regard to the control of black blooms.

## 2. Research Region and Data

**Figure 1:**
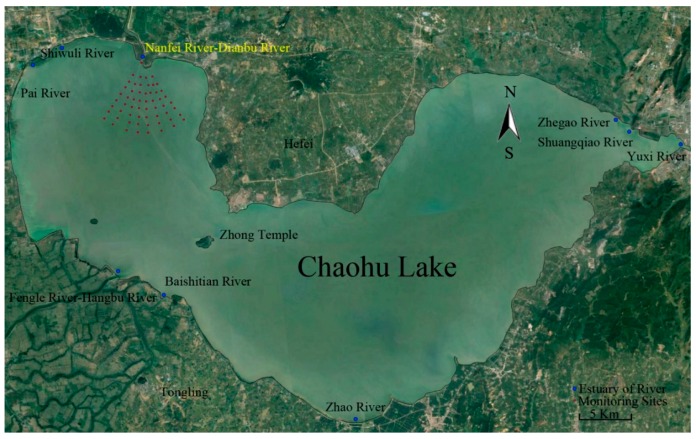
The region and monitoring sites of Chaohu Lake.

Chaohu Lake lies on the left bank of the Middle and Lower Yangtze Plain. It is the fifth largest freshwater lake in China, with an area of approximately 780 km^2^. The lake is shallow, and the average depth is about 4 m. Figure 1 depicts the Chaohu Lake region.

As can be seen in Figure 1, about 33 rivers, including 11 main rivers, exist around Chaohu Lake, and the Yuxi River that lies in the westernmost area is the only river that flows out of the lake. Nanfei River, which is located to the north, is the largest pollution source from the Heifei city, so black blooms are often observed in the estuary of this river in the summer [14,26].

The sampling was conducted in July 2013, when the black blooms are often observed in this area [26,27]. There were 58 monitoring sites with a fan-shaped distribution around the estuary of Nanfei River (red dots, Figure 1). The concentrations of Fe(II) and S(-II) in the water column were measured using phenanthroline [31,32] and the methylene blue spectrophotometric method [32,33], respectively. The TN, TP, soluble phosphorus (SP), CHLA, O_2_ demand (COD_Mn_), and SSC were measured by methods previously reported [32]. The DO, oxidation reduction potential (ORP), pH (National Bureau of Standards scale), and water temperature (TEMP) were determined using a multi-parameter water quality analyzer (U-53, Horiba, Kyoto, Japan). The water depth to the sediments (DEP) and the water velocity (VEL) were also determined using ADV (acoustic doppler velocimetry) with a surveying rod (YSR-1, YSI, Yellow Springs, USA).

## 3. Methods

The sampling was conducted once, and 58 sites were surveyed for 14 factors. Since the model also includes interactions among these factors, the number of parameters largely exceeded the sample size, making ordinary least squares (OLS) estimation ill-defined, calling for sparsity-inducing methods such as the least absolute selection and shrinkage (LASSO) introduced by Tibshirani [30]. Bayesian implementations of the LASSO [34,35,36,37,38] have proved particularly valuable in handling oversaturated models. In this study we used the Bayesian LASSO and resorted to the empirical Bayes method (e.g., [35,39]) for parameter estimation.

Compared to the regression solved by the OLS method, LASSO adds L1-norm of the vector of regression coefficients (i.e., the sum of the absolute values of all components) as a penalty (the L1 penalty) to the negative log-likelihood or equivalently subtract the L1-nrom of the vector of regression coefficients to the log-likelihood. The L1 penalty forces most of the irrelevant effects (the coefficients of redundant predictors) to shrink toward zero, with some of them being set to zero, thereby allowing LASSO to select out the relevant predictors for dependent variables [29]. The regression model fitted by OLS is

(1) Y=μ1+Xβ+εY=μ1+Xβ+ε

Where **X** is the *n* × *p* matrix containing *p* independent variables for *n* samples, **β** is the *p*-vector of regression coefficients, **Y** is the *n* × 1 vector of response values, *µ* is an intercept, **1**. is the *n*-vector of ones, ε is the *n*-vector of zero-mean random errors assumed to be Gaussian. In OLS, the parameters *µ* and **β** are estimated by maximizing the log-likelihood function with regard to these parameters, whereas LASSO estimation requires the maximization of the log-likelihood subject to the L1 penalty. That is

(2) $$\hat{\beta }=\underset{\beta }{argmax}[logL(y|x,\mu ,\beta )-\lambda {\left\|\beta \right\|}_{1}]$$

where $\hat{\beta }$ is an estimation of **β**; *L* is the likelihood function; *y* and *x* are samples of **Y** and **X**, respectively; ‖β‖_1_ is the *l*_1_ norm of **β**; and the regularization parameter λ ≥ 0 determines the amount of shrinkage. This results in $\hat{\beta }$ equaling the OLS regression coefficients when λ = 0 whereas a large enough λ will lead to some of the regression coefficients being zero.

From a Bayesian perspective, the LASSO estimates can be regarded as the posterior modes of the regression coefficients *β*_j_ in Equation (1) under the following hierarchical prior [34,36]

(3) $$\beta_{j}\sim N\left(0,\sigma_{j}^{2}\right)$$

and the Probability Density Function (PDF) of *σ*_j_ can be

(4) $$f\left(\sigma_{j}\right)=\lambda exp(-\lambda \sigma_{j}^{2})\;f(\sigma_{j})=\lambda \exp(-\lambda \sigma_{j}^{2})$$

From a Bayesian perspective, Equations (3) and (4) are equivalent to assigning independent Laplace or double exponential priors on *β*_j_ [37,38]. That is, for a given λ, the two-level prior distribution equal to the prior PDF of *β*_j_ should be as follows:

(5) $$p\left(\beta_{j}\right)=\sqrt{\frac{\lambda }{2}}exp(-\sqrt{2\lambda }\left|\beta_{j}\right|)$$

The two prior levels can be expanded to three levels for forcing the numbers of coefficients to zero and only retaining variables with a relatively large signal-to-noise ratio (SNR) [35].

(6) λ~gamma(a,b)

Where *gamma*(*a*,*b*)is the gamma distribution with shape parameter *a* and inverse scale parameter *b*. When the parameters are set to be small, the prior for λ is essentially non-informative, which can avoid the manual error in parameter selection. The posterior of *β*_j_ should be Gaussian with no explicit form [40,41] in the LASSO method.

The hyperparameters *a* and *b* can be determined using the cross-validation method. The determination coefficients (*R*^2^) and the adjusted determination coefficients (Adjust *R*^2^) are provided for each equation in this paper. Fast empirical Bayesian (EB) method [42], not Markov chain Monte Carlo (MCMC) approach, was employed to fit Bayesian LASSO model and determine the posterior distribution of the model parameters for avoiding the computational burden of MCMC. The t-statistics was used to determine the significance of the *p*-value as OLS regression followed the original algorithm [42]. The greedy coordinate descent algorithm was the numeric method applied to optimize the parameters in EB method [42]. The estimation of parameters successively optimized the objective function of each parameter with the others fixed, which was cycled repeatedly until convergence. The R [43] package EBglmnet was used in this paper to complete the estimation of parameters and the default prior in this package was also used.

## 4. Results and Discussion

As shown in Table 1, the concentrations of TN, TP, and COD_Mn_ were very high. The abundance of nutrients and favorable water TEMP provided a good environment for algae growth. The minimum value of CHLA, which was higher than 20 μg/L, also showed that algae accumulated in the bay. Algae supply rich organic matter for black bloom creation after they die.

The appearance of high concentrations of Fe(II), and S(-II) was in agreement with the observed phenomenon in black bloom water in Taihu Lake [18] and in laboratory controlled experiments [24,25].The ORP implied that weak oxidizing ability in the water was beneficial in maintaining Fe(II) and S(-II). However, the DO was not very low and the water pH was higher than 7, in which the Fe(II) will be oxidized quickly, so that the black bloom disappear quickly after sampling was over. The concentrations of SSC were very high. The large standard deviation (SD) of SSC implied that the concentration gradient was large, and an edge of the black bloom will be clearly distinguished. This is the reason why black bloom should be called a “block” [1] or “spot” [24,44].

Pearson’s correlation coefficients between Fe(II), S(−II), SSC, and other environmental factors are shown in Table 2. All variables were mean-centered and scaled to unit variance to remove the impact of different units. The results show that the correlations among Fe(II), S(−II), and SSC was stronger than correlations among other variables. The combination of correlation result, high values of Fe(II) and S(−II) shown in Table 1 and the low solubility product of FeS indicated that the granules in water were formed by FeS precipitation, as previous research has shown [17,19,26]. The results also suggested, because FeS precipitation is black, that high concentrations of FeS caused the water to become so turbid that it appeared “black”.

The TN, TP, SP, and COD_Mn_ had positive correlations with Fe(II), S(-II), and SSC with a *p* < 0.05. It was shown, as in former studies [11,17,45], that the nutrients and oxygen-consuming substances are important for causing black blooms in lake bays. The DEP was the only variable with all three factors. This is most likely due to the source of the sulfide and Fe(II) being the underlying sediments and thus the measurements in the lake reflect the diffusion and mixing of these species in the water column.

It was surprising that DO and ORP showed no significant correlations with Fe(II), S(−II), or SSC. This might have been because the lake water quality was undergoing recovery during sampling. The depth of the water was shallow (Table 1) and reoxygenation of water proceeded quickly. The quick disappearance of the black bloom after sampling completely supported this hypothesis. Another reason may be that the diffusion and mixing of these factors in water column influenced the relationships. The interaction and uncertainty among variables relationships, which cannot be directly measured by Pearson correlations, may be the third explanation for these seemingly counterintuitive relationships. It also showed the necessity to calculate the Bayesian LASSO regression for interaction estimation and determining the uncertainty of relationships.

Although the reaction between Fe(II) and S(-II) controls the concentration of FeS in the water column [46], S(−II) and SSC were excluded from the predictors when Fe(II) was the response variable due to the strong correlations between them and Fe(II), and all other factors would be excluded from regression if they were included. For S(−II) as the response variable, Fe(II) and SSC were excluded for the same reason. Both Fe(II) and S(−II) were considered in the regression for SSC. Therefore, there were 11 main putative and ½ × 11 × 10 = 55 interaction predictors in regression models for Fe(II) and S(−II). There were 13 main putative and 65 interaction independent variables for SSC as the response variable.

Table 3 shows the optimal hyperparameters selected by mean square errors (MSEs) and standard errors (SEs) in cross-validations. The cross-validations were conducted using five folders. The prior distributions for λ in all regression equations are given as *gamma(0.1,0.1)*. The values of optimal hyperparameters *a* and *b* were not important for application in this area. However, the small values indicated that the variables selection with non-informative priors was successful.

The explanation ratio (ER) of the i-th factor in regression equations can be determined by its coefficient as Equation (7).

(7) $$ER_{i}=\frac{\left|\beta_{i}\right|}{\sum_{i}\left|\beta_{i}\right|}$$

The regression equation selected from the 66 candidate variables for Fe(II) is as follows:

*Fe(II)* = 0.959 × *TP* + 0.134 × *TN* × *DO* + 0.167 × *ORP* × *CHLA* + 0.0653 *R*^2^ = 0.841, *Adjust R*^2^ = 0.832, at *α* = 0.05 *significance level* (*p* < 0.05) (8)

All terms in Equation (8) were significant (*p* < 0.05). Both the *R*^2^ and adjusted *R*^2^ of the equation were lager than 0.80, which showed that the efficiency of the regression equation was satisfactory. The Bayesian LASSO model selected the three most important terms to investigate the usefulness of the method in extracting key environmental factors for concentrations of Fe(II).

The LASSO model selected the TP, TN × DO, and ORP × CHLA as the three most important factors for the concentration of Fe(II) in black blooms. Consistent with the correlation analysis, TP was the most important factor and showed about 76.1% ER, as its coefficient indicated. SP, which was also one of the candidate factors, was ignored in the LASSO equation. SP also showed a significant correlation with Fe(II) in terms of the Pearson coefficient; however, it should be excluded from the equation by the LASSO method due to its significantly less important and strong collinear relationship with TP. Chemically thinking, the reduction of iron oxides in the lake will release adsorbed phosphate and the relationship between TP and Fe(II) may be partly related to this rather than just to SP in the water column.

The TN × DO interaction explained about 10.6% of Fe(II) presence. Compared with the correlations results, this indicates that the interaction of TN and DO, and not among themselves, has significantly positive effects on Fe(II). This is quite different from the results obtained in a steady state [47,48]. Three reasons may explain this finding. First, the sampling sites were in a lake bay where the water dynamic condition was far from a steady state. DO was high due to reoxygenation, so the occurrence of the black bloom reflects the fact that DO did not affect the existence of Fe(II) for a short time in shallow (Table 1) lake bay. Second, the oxidation reaction would have reduced the concentration of Fe(II) for a period of time. It was hard to observe the DO effect from the cross-sectional dataset used in this study, which is also a possible reason for the non-significance relationship between DO and Fe(II). Third, as shown in Table 2, significantly negative correlations were found between TN and DO. The linear regression shows that the coefficient of DO was −0.2 for TN as the response variable, whereas the coefficient of TN was 0.51 for Fe(II) as the response variable. Combining these results, Equation (8) indicated that Fe(II) may have decreased when DO increased because TN also declined.

**Figure 2:**
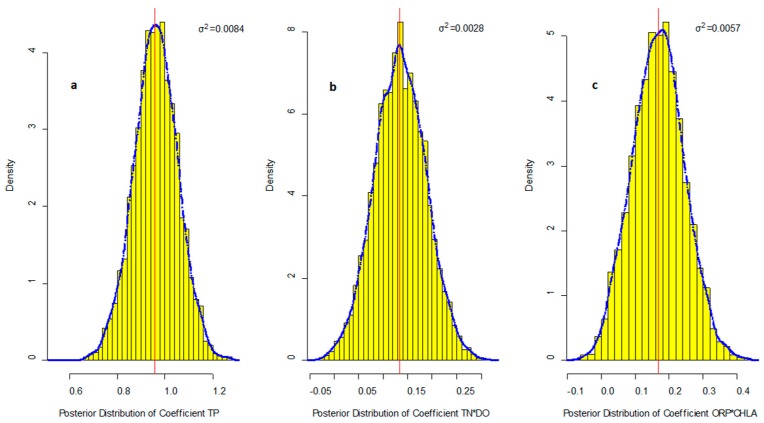
The posterior distribution of coefficients for Fe(II). The blue dotted line is the kernel density estimation for the coefficients, the solid red line is the posterior mean value of coefficients, σ^2^ is the posterior variance of coefficients, and yellow bars are the histogram of the density of posterior coefficients; (**a**) total phosphorus (TP) coefficient; (**b**) TN × dissolved oxygen (DO) coefficient; (**c**) oxidation reduction potential (ORP) × chlorophyll a (CHLA) coefficient.

Both ORP and CHLA had no significant relationships with Fe(II), whereas ORP × CHLA showed about 13.3% ERs in Equation (8). It may be because the decomposition of dead algae would consume oxide substance in the water [1] and decreased the ORP to obstruct the ferrous oxidation. Although aeration was the most popular action to control the Fe(II) concentration, the importance of ORP × CHLA and TN × DO clarified that aeration combined with the TN and algae reduce management would be more effectively for reducing the Fe(II) concentration in shallow bay. This finding also partially cleared the doubt about the weak correlations between Fe(II) and CHLA and ORP as measured by Pearson’s coefficients. The poster distributions and variance of coefficients in Equation (8) are shown in Figure 2.

The coefficients of TP exhibit the lowest posterior uncertainty with coefficient of variation (CV) 0.10. The posterior uncertainty of the ORP × CHLA interaction effect on Fe(II) was higher than that of interaction effect of TN × DO. The CV of the two interaction effects were 0.45 and 0.39, respectively. Both TN × DO and ORP × CHLA had a small probability that the coefficients of them in Equation (8) would be negative. This reflected the interaction effects of TN and DO, ORP and CHLA might also have a negative relationship with Fe(II) for small probability although the mean effect of these interactions should be positive.

Equation (9) shows the relationships between S(−II) and environmental factors, for which all terms were significant at the *p* < 0.05 level.

*S(−II)* = 0.535 × *TP* − 0.325 × *TP* × *DEP* + 0.421 × *TP* × *Ph* + 0.170 × *TP* × *COD_Mn_* − 0.0128 *R*^2^ = 0.628, *Adjust R*^2^ = 0.602. at *α* = 0.05 *significance level* (*p* < 0.005) (9)

Although the goodness of fit was not as good as in Equation (8), it was acceptable. The regression was more complex than for Fe(II). Like in Equation (8), TP was the only single aquatic factor that was selected. Although the ER of single TP decreased to 37.0%, it still had the largest explanation of all items. TP emerged at each item, also showing that phosphorus plays a key role in S(−II) production.

The role of oxygen for S(−II) was not as important as for Fe(II). Neither DO nor ORP were involved in the regression. This was in agreement with the results of the correlation coefficients, which indicated that the aeration method alone was not a suitable method to control the concentration of S(−II). The interaction of DEP and TP showed that TP distribution in the water had an obvious impact on decreasing S(−II). It also suggested that water depth was an important factor for S(-II). This is easy to explain considering the shallow DEP of sampling sites and the fact that much S(-II) in water was released from sediment [14]. The pH and COD_Mn_ in the remaining two items showed that the effect of TP on S(−II) would be affected by them.

**Figure 3:**
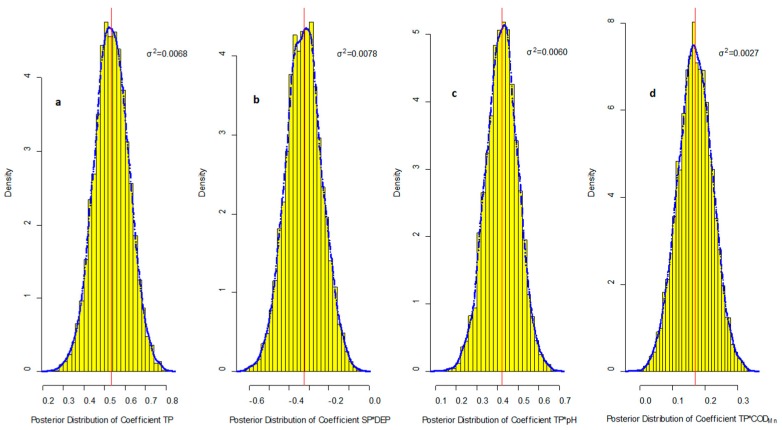
The posterior distribution of coefficients for S(-II). The blue dotted line is the kernel density estimation for the coefficients, the solid red line is the posterior mean value of coefficients, σ^2^ is the posterior variance of coefficients, and the yellow bars are histogram of density of posterior coefficients; (**a**) TP coefficient; (**b**) TP × water depth (DEP) coefficient; (**c**) TP × pH coefficient; (**d**) TP × O_2_ demand (CODMn) coefficient.

Figure 3 shows the posterior distribution of coefficients for S(-II). Different from the results of Fe(II), almost all items had a fixed positive symbol and showed no doubt about these interaction effects on S(−II). The CV of these items were about 0.15, 0.27, 0.18, and 0.31. Although posterior the uncertainty of the TP coefficient is higher than in the regression equation for Fe(II), it was still the lowest in these relationships which suggest that controlling the TP concentration is also a reliable method to reduce the S(−II) pollution.

Equation (10) was used to perform the regression for SSC, and all predictors turned out to be significant at α = 0.05 significance level (*p* < 0.05)

*SSC* = 0.327 × *Fe*(*II*) + 0.246 × *S*(−*II*) + 0.147 × *TP* − 0.064 × *DEP* − 0.347 × *Fe*(*II*) × *DEP* − 0 *R*^2^ = 0.796, *Adjusted R*^2^ = 0.778, at *α* = 0.05 *significance level* (*p* < 0.05) (10)

Although the goodness of fit for Equation (10) was a little worse than for Fe(II), it was better than Equation (9). The high ER values, which were about 28.9% and 21.8% of Fe(II) and S(−II) terms, respectively, suggested that SSC was constituted from FeS. With 12.9% ER, TP emerged as an important predictor of the onset of SSC. In contrast with Fe(II) and S(−II), the item with DEP only was selected out by LASSO for 5.6% ER. This ratio increased to 30.7%, which was the highest in all items in Equation (10), for the Fe(II) × DEP interactions. Like the regression equation for S(−II), this indicated the importance of diffusion from sediment in SSC because of shallow depths in the lake bay.

**Figure 4:**
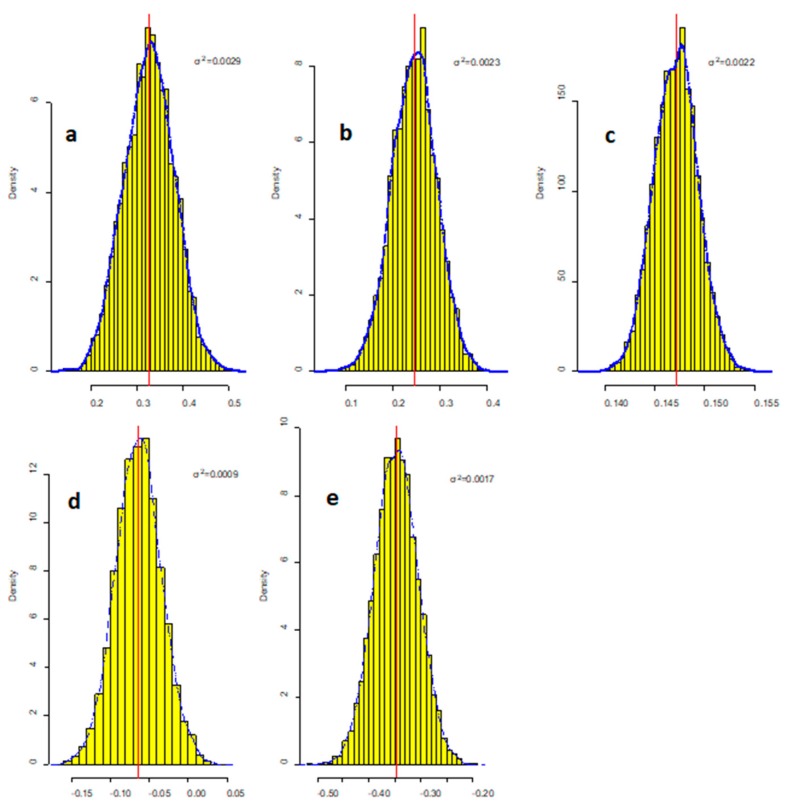
The posterior distribution of coefficients for SSC. Blue dotted line is the kernel density estimation for the coefficients, solid red line is the posterior mean value of coefficients, σ^2^ is the posterior variance of coefficients, and yellow bars are histogram of density of posterior coefficients; (**a**) Fe(II) coefficient; (**b**) S(-II) coefficient; (**c**) TP coefficient; (**d**) DEP coefficient; (**e**) Fe(II) × DEP coefficient.

Figure 4 shows the posterior distribution of coefficients for SSC. The highest uncertainty measured by the CV was DEP at 0.47. Though the coefficient of DEP was negative, the existence of a positive value area in posterior distribution of its regression coefficient also corroborated the uncertainty effect of DEP. These indicated that the effect of deposition for reducing SSC might be influenced by other unobserved aquatic factors which should be researched in future. The highest coefficient of Fe(II)×DEP and its lowest CV in all items of Equation (10) showed that this interaction was the most important variable that influenced SSC. It suggested the source of SSC in black bloom was resuspension from sediment which were also showed by references [11,17].

The CV between Fe(II) and SSC, S(−II), and SSC was low, implying that SSC in the bay was more likely formed by FeS. In contrast with the results of Fe(II) and S(−II), the CV of the effect of TP on SSC was large, which is not surprising since TP cannot form granules directly. However, the posterior distribution of the regression coefficient showed that the higher TP leading to higher SSC was a fixed factor.

## 5. Conclusions

Black blooms are a serious and complex problem in lake bays. Fe(II), S(-II), and SSC are the key elements that should be considered when attempting to remediate black blooms. In this paper, the correlations among Fe(II), S(−II), SSC, and 11 environmental variables were estimated in the Chaohu Lake bay. The Bayesian LASSO regression method was also employed to successfully select important factors from many variables for Fe(II), S(−II), and SSC. The regression equations for these factors in a black bloom in a lake bay were obtained and showed good agreement with the observations. The results confirmed that the SSC was constituted from FeS. TP, which had about 76.1%, 37.0%, and 12.9% ERs in Fe(II), S(−II), and SSC regression equations, was the single key environmental variable for controlling the concentrations of Fe(II), S(−II), and SSC.

The results showed interactions between environmental factors under consideration, which cannot be estimated from correlations, also play an important role in prediction of Fe(II), S(-II), and SSC. TP emerged at each interaction item of the regression equation using S(-II) as the response variable. For Fe(II), factors related to oxygen and algae activity, such as TN × DO (10.6% ER) and CHLA × ORP (13.3% ER) were also important interaction variables. This implied that the effects of oxygen and algae on Fe(II) are complex but obviously at a shallow lake bay. DEP was not only the major factor in the interaction items (30.7%) but was also an important single factor (5.6% ER) for SSC, indicating the importance of diffusion for the SSC controlling because most of it were suspension from sediments at a shallow bay.

The uncertainty of relationships is reflected in the spread of the posterior distributions and CV of coefficients. The posterior distribution of some regression coefficients, such as the coefficient of TN × DO, showed these factors might have both negative and positive effect on Fe(II) and SSC. It implied that these relationships should be considered carefully. However, the uncertainty of TP coefficients was low and there was no doubt about its effect as inferred from its posterior distribution for all regressions.

In summary, the interactions between environmental factors proved to be important for the onset of the black blooms. Reducing the concentration of TP would be the most effective method for managing black blooms. However, the effects of other factors might depend on the environmental background of the lake bay. This study provides a useful exploration for controlling black bloom in lakes and developing management policy. Further research is required for the quantitative analysis of relationships among aquatic factors of black bloom in other lakes.

## Figures and Tables

**Table 1:** Statistics of observations (*n* = 58).

Aquatic Factors	Maximum Value	Mean Value	Minimum Value	SD
Fe(II) (mg/L)	5.51	1.27	0.24	0.96
S(-II) (mg/L)	0.15	0.04	0.02	0.02
SSC (mg/L)	1188.00	114.41	4.00	192.45
TN (mg/L)	4.84	2.17	0.33	1.18
TP (mg/L)	1.48	0.37	0.09	0.27
SP (mg/L)	0.42	0.11	0.02	0.08
DO (mg/L)	8.43	6.88	3.84	0.99
CHLA (μg/L)	170	53	21	31
COD_Mn_ (mg/L)	110.86	77.96	68.76	7.06
ORP (mV)	168	131.13	73	25.33
TEMP (℃)	28.80	25.35	23.80	1.84
DEP (m)	1.7	1.21	0.9	0.15
VEL (cm/s)	56.00	11.04	1.20	8.05
pH	8.35	8.04	7.22	0.24

**Table 2:** Pearson correlation coefficients.

Aquatic Factors	Fe(II)	S(−II)	SSC	TN	TP	SP	DO	CHLA	COD_Mn_	ORP	TEP	DEP	VEL	pH
Fe(II)	1													
S(−II)	0.72 **	1												
SSC	0.87 **	0.83 **	1											
TN	0.50 **	0.41 **	0.49 **	1										
TP	0.76 **	0.68 **	0.80 **	0.78 **	1									
SP	0.39 **	0.26 *	0.37 **	0.62 **	0.73 **	1								
DO	−0.18	0.05	−0.04	−0.40 **	−0.34 **	−0.51 **	1							
CHLA	−0.03	0.04	0.02	0.23	0.08	−0.04	0.18	1						
COD_Mn_	0.35 **	0.41 **	0.41 **	0.58 **	0.58 **	0.52 **	−0.27 *	0.20	1					
ORP	−0.21	0.07	−0.05	−0.26 *	−0.09	0.07	0.24	−0.03	−0.12	1				
TEP	−0.15	−0.06	0.00	−0.15	−0.09	0.07	−0.13	0.04	0.04	0.37 **	1			
DEP	−0.36 **	−0.28 *	−0.34 **	−0.27 *	−0.38 **	−0.17	0.07	0.03	−0.24	0.38 **	0.20	1		
VEL	−0.02	0.02	0.00	−0.03	0.06	0.12	0.11	0.00	−0.11	0.44 **	0.18	0.14	1	
pH	−0.15	0.14	0.06	−0.20	−0.11	−0.17	0.68 **	0.10	−0.24	0.67 **	0.20	0.24	0.33 **	1

** significant at *p* < 0.01; * significant at *p* < 0.05.

**Table 3:** Optimal parameters.

Response Variables	Optimal Hyperparameters		Cross Validation Criterions	
	*a*	*b*	MSE *	SE **
Fe(II)	−0.60	0.50	0.45	0.40
S(-II)	−0.10	0.50	0.94	1.46
SSC	0.01	0.10	0.20	0.18

* MSE: mean square error. ** SE: standard error.
